# Training for impact: the socio-economic impact of a fit for purpose health workforce on communities

**DOI:** 10.1186/s12960-016-0143-6

**Published:** 2016-08-15

**Authors:** Björg Pálsdóttir, Jean Barry, Andreia Bruno, Hugh Barr, Amy Clithero, Nadia Cobb, Jan De Maeseneer, Elsie Kiguli-Malwadde, André-Jacques Neusy, Scott Reeves, Roger Strasser, Paul Worley

**Affiliations:** 1 Training for Health Equity Network, New York, United States of America; 2 Consultant Nursing and Health Policy, International Council of Nurses, Geneva, Switzerland; 3 International Pharmaceutical Federation, The Hague, Netherlands; 4 Centre for the Advancement of Interprofessional Education (CAIPE), London, United Kingdom; 5 Family and Community Medicine, University of New Mexico School of Medicine, Albuquerque, New Mexico United States of America; 6 Office for the Promotion of Global Healthcare Equity, Division of Physician Assistant Studies, University of Utah School of Medicine, Salt Lake City, Utah United States of America; 7 Department of Family Medicine and Primary Health Care, Ghent University, Ghent, Belgium; 8 The Network: Towards Unity for Health, Ghent, Belgium; 9 African Center for Global Health and Social Transformation (ACHEST), Kampala, Uganda; 10 The Training for Health Equity Network, New York, United States of America; 11 Interprofessional Research, Centre for Health and Social Care Research, Kingston University and St George’s, University of London, London, United Kingdom; 12 Northern Ontario School of Medicine, Lakehead and Laurentian Universities, Sudbury and Thunder Bay, Canada; 13 Flinders University, Adelaide, Australia

**Keywords:** Health workforce education, Economic impact, Social impact, Social accountability, Social determinants of health, Distributed learning, Community engagement, Interprofessional education, Primary care

## Abstract

Across the globe, a “fit for purpose” health professional workforce is needed to meet health needs and challenges while capitalizing on existing resources and strengths of communities. However, the socio-economic impact of educating and deploying a fit for purpose health workforce can be challenging to evaluate. In this paper, we provide a brief overview of six promising strategies and interventions that provide context-relevant health professional education within the health system. The strategies focused on in the paper are:

1. Distributed community-engaged learning: Education occurs in or near underserved communities using a variety of educational modalities including distance learning. Communities served provide input into and actively participate in the education process.

2. Curriculum aligned with health needs: The health and social needs of targeted communities guide education, research and service programmes.

3. Fit for purpose workers: Education and career tracks are designed to meet the needs of the communities served. This includes cadres such as community health workers, accelerated medically trained clinicians and extended generalists.

4. Gender and social empowerment: Ensuring a diverse workforce that includes women having equal opportunity in education and are supported in their delivery of health services.

5. Interprofessional training: Teaching the knowledge, skills and attitudes for working in effective teams across professions.

6. South-south and north-south partnerships: Sharing of best practices and resources within and between countries.

In sum, the sharing of resources, the development of a diverse and interprofessional workforce, the advancement of primary care and a strong community focus all contribute to a world where transformational education improves community health and maximizes the social and economic return on investment.

## Background

There is increasing awareness that to maximize positive impact on health, education systems need to be embedded in health systems. The recent Ebola epidemic in West Africa is a grim reminder that not investing in a fit for purpose workforce deployed at all levels of a health system can devastate health, cripple development and have global repercussions [1, 2]. The challenge is urgent. The 2013 WHO report estimates that the shortage of healthcare workers is likely to increase from 7.2 million to 12.9 million by 2035 [3]. Producing and maintaining an adequate supply is not the only existing challenge. There is a misalignment between health system needs and the education and training of the health workforce [4]. Chisholm and Evans estimate that inefficiencies such as inappropriate skill mix, attrition, retention issues and mal-distribution of health workers; unnecessary or too long hospital admissions and inappropriate training could be costing the global health economy more than US$500 billion [5]. These challenges are major impediments to universal health coverage and health equity in high-, middle- and low-income countries. Producing a context-relevant workforce that is fit for purpose requires education to be designed accordingly.

The answer to how can the impact of educational investments be maximized may be socially accountable education. It is increasingly seen as a mechanism to maximize impact and is included in several major policy guidelines and documents such as the World Health Organization’s *Guidelines on Transforming and Scaling up Health Professional Education*. Socially accountable (SA) health professional education is broadly defined as “the obligation to direct their education, research and service of activities towards addressing the priority health concerns of the community, region and/or nation that they have a mandate to serve. The priority health concerns are to be identified jointly by governments, healthcare organizations, health professionals and public”. It means ensuring learners understand the culture, the needs and the assets of the communities in which they learn and then develop relevant competencies for practicing in these communities, becoming a more “fit for purpose” worker. Strategies to create a fit-for purpose workforce include giving community members a voice in both the selection and training of students, training diverse students early on and throughout their curriculum in primary care and underserved settings, training a cadre of health workers including community health workers, using competency-based assessment of theoretical learning, integrating and reinforcing learning on the effect of social determinants of health and equity on both individuals and populations, and learning to practise in interprofessional teams.

**Fig. 1:**
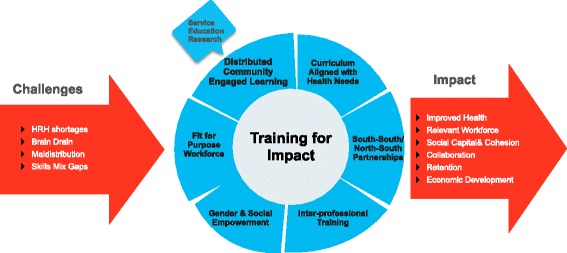
Training for impact. This figure illustrates the contributing factors to health workforce shortages, strategies to utilize during health professional education and the impacts on communities when the strategies are deployed

Collaboration and sharing of best practices to evaluate the impact of such complex dynamic educational interventions is challenging, but methodologies are evolving to better capture the impact of SA and other transformative education efforts [6–9]. Illustrative examples, some of which are presented below, from across the globe suggest that a combination of strategies can contribute to improved workforce and health outcomes which may persuade key decision makers that investment in education generates a sound return on investment (see Fig. 1). In this paper, we provide a brief overview of six promising strategies and interventions, which are potentially important links in a chain of evidence (see Table 1).

**Table 1:** Terms of reference: strategies and abbreviations. This table clarifies terms and abbreviations used within this article

Terms of reference: strategies and abbreviations
Distributed, community-engaged learning: the health and social needs of targeted communities guide education, research and service programmes with input and active participation from the communities served. Education occurs in or near underserved communities using a variety of educational modalities including distance learning.
Fit for purpose workers: education and career tracks are designed to meet the needs of the communities served. This includes cadres such as community health workers, accelerated medically trained clinicians, nurse practitioners and extended generalists.
Gender and social empowerment: ensuring a diverse workforce that includes women have equal opportunity in education and are supported in their delivery of health services.
Interprofessional training: teaching the knowledge, skills and attitudes for working in effective teams across professions.
South-south and north-south partnerships: sharing of best practices and resources within and between countries.

### Distributed community-engaged learning

Community-engaged education builds on and strengthens social capital, defined by Robert Putnam as: “(…) connections among individuals in social networks and norms of reciprocity and trustworthiness that arise from them” [10]. It involves interdependent and reciprocally beneficial partnerships between education institutions and the communities they serve. Integrated health and education systems can increase impact and reduce costs by tapping into social capital, local human resources and community assets [11, 12].

Community engagement is a fundamental part of setting a schools’ education, research and community development mission. It seeks to address power inequities between academies and their community partners, supporting the alignment of communities’ healthcare needs with student learning objectives and activities and encouraging students to develop skills and motivation to practise in specific community settings. Communities directly contribute to students’ learning of local social determinants of health and equity. Community-engaged education is broadly consistent with the World Health Organisation’s model of social accountability, recommended in its guidelines on transforming and scaling up health professional’s education and training [13, 14]. Further, distributed community-engaged learning, whereby the entire community is the campus and the community is actively involved in student education, expands its scope and impact. Learning is distributed throughout this “campus” using both immersion experiences and distance technologies. This type of health workforce education can result in direct economic benefits to rural and underserved communities, even before the students graduate.

#### Strategy

Distributed community-engaged learning.

#### Example

The Northern Ontario School of Medicine (NOSM) engaged its communities in the development of its academic programmes from the outset, starting with a curriculum workshop and continuously involving a wide range of community partners, including members of local Aboriginal and Francophone communities. Through distributed community-engaged learning, community members play a vital role: in selecting students for the 4-year undergraduate medical programme, by participating as standardized patients, and by providing local support for students during their community placements including the eight month comprehensive community clerkship where they learn their core clinical medicine from a family practice and community perspective [15].

#### Outcomes

Ninety-four percent of NOSM medical graduates who have undertaken their postgraduate training with NOSM are practicing in the region, including 33 % in remote rural communities. The School has had a substantial socio-economic impact including the following: new economic activity, more than double the School’s budget; enhanced retention and recruitment for the universities and hospitals/health services; and a sense of empowerment among community participants. The total economic contribution to 62 communities in Northern Ontario was estimated at $67.1 million based on $36.3 million in spending by NOSM and $1.0 million spent by students [16].

#### Strategy

Rural community-based education.

#### Example

In Australia, universities have been encouraged to move the location of health workforce education from cities to underserved rural communities. Medical schools now have 25 % or more of their students undertaking an entire clinical year based in rural communities. They also have a target of admitting at least 25 % of their students from rural communities.

#### Outcomes

This has resulted in significant government investment directly into rural and remote communities totalling AUD129 million for the financial year 2013/2014. This covers infrastructure, staffing and support for students, with likely spin-off benefits for job creation and economic benefit as demonstrated at NOSM [17]. It has also benefited rural clinicians who have become faculty and increased the clinical workforce in rural and remote areas. This has improved quality of care, reduced professional isolation and resulted in increased job satisfaction [18]. Early evidence suggests that over 50 % of students chosen and educated in this way choose rural careers, resulting in further economic benefit to rural communities [19].

### Curriculum aligned with health needs

Health workforce education institutions with a social accountability mandate design their curricula to address the needs of the communities and regions they serve.

#### Strategy

Curriculum focus on regional priority health needs.

#### Example

The Ateneo de Zamboanga School of Medicine (ADZU) in the Philippines operates on similar principles as NOSM but with much greater resource constraints. Its curriculum is designed to meet priority health needs in the region. Community engagement is a continuous theme throughout the 4-year programme with students undertaking community development projects and obtaining much of their clinical learning in small rural communities commencing in year 1 and culminating with students living in the same small communities for the entire fourth year.

#### Outcomes

These student-initiated projects are solving major health issues in the community, drawing heavily on the social capital available. Outcomes have included students facilitating improvements in water and sanitation, transportation for pregnant women and school children and other projects, some of which evolved into non-governmental organisations (NGOs). Over 90 % of ADZU graduates continue their training and clinical practice within the region. In addition, the infant mortality rate in this region has declined from 75/1000 in 1995 to 8.2/1000 in 2008, significantly greater than national improvement figures [20, 21].

#### Strategy

Curriculum includes social determinants of health.

#### Example

At the University of New Mexico Health Sciences Center (UNM), USA, funding partners such as the State Government, insurance companies and managed care organizations are seeing the economic and health benefits of UNM’s reallocation of resources “upstream”, focusing on social determinants, a non-traditional area for most at academic health centres. Until recently, such a focus was of interest mostly in progressive corners, bringing no economic reward to institutions. Even for-profit insurance companies now recognize the value of wellness and keeping people out of hospitals and emergency rooms; hence, addressing these determinants suddenly seems “profitable”. Academic health centres can only address them effectively by genuine community engagement and partnerships with other sectors such as public education, agriculture, business and government.

#### Outcomes

This approach has tightened the link between education and clinical service on campus and has led to the hiring, training and incorporation of community health workers (CHW) into health teams and education programmes as field-based, cultural and community-campus brokers, a model adopted from international models. Funding has grown and is now continuous, versus grant-based, as the effectiveness of these models of service and education is emerging [22].

### Fit for purpose workers

Achieving universal health coverage requires a fit for purpose workforce with the competencies needed to address priority health issues in each country or region [23]. To address massive shortages of healthcare providers in underserved areas in a cost-effective way, many countries have established different types of healthcare workers, such as nurse practitioners and accelerated medically trained clinicians (AMTCs). They become key members of the healthcare team, increasing access to care. Because of different regionally based needs, the education, title and regulations vary; thus, health systems and health policy have not always maximized their use. AMTCs are regionally specific clinicians trained under the medical model in an abbreviated/accelerated manner, which enables them to be cost effective and malleable based on the skill mix need. They are often cited as the frontline of primary care as they serve largely marginalized and rural populations. Examples of this cadre date back to the 1650s in Russia with the Feldshers and include clinical officers, physician assistants, health officers and medical assistants, among others. Malawi’s medical assistants (established 1890s) and clinical officers (established 1979) have been the backbone of healthcare, as the government created them to serve at the community level in primary care, and district level in surgery. Malawi opened its first medical school in 1991 and in 2009 was graduating approximately 60 physicians per year. The challenge in attracting physicians to Malawi and retaining them after graduation, let alone deploying them to rural areas, is a continuous struggle. The Health Sector Plan 2011–2016 strongly endorses their AMTCs [24].

CHWs and other community health volunteers such as accredited social health activists (ASHAs) in India have evolved to help communities’ link into the healthcare chain, increasing early identification, tracking, treatment and referrals. India has expanded ASHAs by 700,000 to study their performance. They were found to be mostly (>80 %) engaged with home visits, escorting for deliveries, antenatal, breastfeeding and immunization counselling. They were also active in community activism for health, TB medication provision and engaging with care providers of ill children. Their expansion is under India’s National Rural Health Mission Programme [25].

Campbell et al. (2015) note that a “paradigm shift in health workforce development efforts, moving towards a more diverse range of skills supporting primary care” will need to occur in line with the 2030 global workforce strategy. Community-based cadres not previously included in studies will need to be at country/regional levels to be able to expand the recruitment, education, support and retention of these workers. Defining and categorizing these workers will aid in the education, regulation, scope of practice and clarity in stronger services provided [26].

In Bangladesh, a value for money assessment of a community-based midwifery diploma program indicated it yielded a 16.2 return on investment, using only the number of caesarean sections avoided as a measure [27].

Investment in primary care provides both economic and health returns on investment for government and communities served [28–30]. Creating primary care-oriented training and new career pathways in rural and remote communities is another strategy employed. In Queensland Australia, Queensland Health’s Rural Generalist Pathway provides a supported training and career pathway for junior physicians to train in rural and remote medicine, combined with financial and professional recognition. Early evidence suggests the strategy is creating its own supply line for rural communities in addition to providing the government with high returns on investment. For example, employing rural generalists with advanced anaesthetic and obstetric skills allowed for local deliveries resulting in a 120 % return on investment in terms of cost savings [31].

### Gender and social empowerment

While strengthening education systems can be expensive, studies from the Organization for Economic Cooperation and Development indicate that individuals and governments receive significant economic and financial returns on investment in higher education [32]. In addition to economic benefits, recruiting and supporting women and other under-represented populations into health workforce education empowers not only individuals but also communities and can generate social, economic and health benefits for others in the form of social capital. An evaluation of a midwifery training addressing maternal health needs of rural Afghanistan empowered women through educating them, helping them contribute to family income and gain economic independence, encouraging female education by prioritizing women’s health needs [33]. Educating women to become health workers can have additional benefits, since according to the International Labour Organisation, poor women are more likely than men to invest in their children’s education and thereby also becoming role models for their children [34].

Research is beginning to emerge showing that when behaviour, attitude and skills of healthcare professionals mirror those of the community they serve, quality increases and disparities in health outcomes decrease [35].

A recent international study demonstrates that student selection strategies to ensure that members of underserved communities can pursue careers in health is not only effective in achieving a fair representation of underserved communities within the student body but is likely to increase the number of graduates working with underserved populations [36]. For example, the stepladder programme at the University of the Philippines, Manila–School of Health Sciences, is a sequential and continuous curriculum designed to produce midwives, nurses and doctors to serve low resource and underserved communities. The programme draws on and strengthens social capital in disadvantaged communities in the Philippines. Students, the School and the sponsoring community have a social contract ensuring that students return and serve in their community. The School works closely with the local health department and communities. While this has not been independently evaluated, it is likely that social capital built over time contributes to the high retention rates of more than 80 % of graduates still working in disadvantaged regions [37].

### Interprofessional training

Given the complexity of healthcare and the needs to which it responds, the short shelf life of knowledge and the importance of addressing the range of factors that impact health and wellbeing, there is a compelling need for developing diverse healthcare teams. Interprofessional education (IPE) is a key means by which providers can learn to work together to develop the core competencies required for effective teamwork and collaborative practice [38]. Through the development of a workforce with these competencies, IPE is regarded as an effective approach to providing education experiences to support improvements in the delivery of a range of different health and welfare services. IPE in both undergraduate and postgraduate programmes was one of the 11 recommendations in the WHO evidence-based guidelines on transforming and scaling up health professional education and training [14]. Moi University in Kenya, for example, prepares dentistry, medical, nursing, physical therapy and psychology students for cost-effective collaborative practice in underserved rural communities. Students participated in problem-based and service learning at health clinics and during outreach activities and initiated projects such as helping communities protect water springs [39]. In Namibia, pharmacy students are placed in isolated rural communities, linked to hospitals, where they learn and provide services as part of a team of medical students and pharmacist assistants [40]. By creating a continuum of IPE towards team-based practice, it can support the paradigm shift from a fragmented sub-specialization approach towards a team-based patient-centred one. Reduction in cost and waste of ineffective teams has been documented, although the evaluation of IPE is challenging [41, 42].

### South-south and north-south partnerships

Increasingly, institutions, organizations and communities work together to address the issue of workforce development. This can take the form of collaborative research, sharing tools and experiences, leveraging resources, producing outcomes and accelerating the sharing of knowledge.

The Medical Education Partnership Initiative (MEPI) is a unique example of collaboration that has brought positive change in health professions education through collaboration. Initially, the collaboration was primarily based on north to south partnerships between African and American Universities. However, during implementation, south-south collaboration increased to a scale that had not been documented before in the region. Consortia of health professions education institutions were formed in countries to pool and share resources, creating a unified national approach to transforming education. MEPI has also encouraged engaging other stakeholders like the Ministry of Health, education medical councils and communities to implement the project together [43, 44]. The established schools are helping new schools by training faculty and using grant funds to purchase learning materials for their students. For example, the University of Zambia is sharing its skills lab with two newly formed schools, while in Uganda, the schools share community sites and faculty. In both cases, this has gone beyond government-owned schools and included private for-profit schools [45].

The Nursing Education Partnership Initiative (NEPI) currently in place in six African countries is strengthening education, governance, leadership and administrative capacity of nursing and midwifery institutions and their clinical and academic faculty to produce skilled nurses and midwives. A NEPI advisory group, led by the Ministry of Health in each country, ensures NEPI capacity building efforts are aligned with country efforts, thus maximizing resources as well as addressing multiple needs. NEPI has supported an increase in student intake; introduced competency-based curricula, innovative learning strategies, and academic-service partnerships; strengthened the teaching and learning workforce; and improved infrastructure and adoption of quality standards. To date, NEPI has resulted in increased enrolment in nursing and midwifery in Bachelor of Science in nursing programmes and in Masters programmes with the aim to prepare new faculty to serve in underserved areas. Exchanges with universities in the south and between NEPI-supported countries and institutions have resulted in the implementation of new clinical preceptor programmes, more efficient and creative use of simulation resources for clinical teaching, political endorsement/recognition of the need for clinical nurses, midwives and faculty to be prepared for advanced practice in their area of specialization to educate the next generation of nurses and address current health needs [46].

Another example of south-south cooperation is the Primafamed network. The Network started in 1997 as a cooperation of the eight departments of family medicine and primary healthcare of South Africa. The Network evolved in 2006 towards a Southern and Eastern African network, involving departments of family medicine in Rwanda, Congo, Tanzania, Kenya and Uganda. In 2009, the “Twinning Project” developed an original cooperation strategy, whereby a South African department of family medicine would “twin” with a region or a country, where there are limited training capacities, sometimes even not a medical faculty or university [47].

The International Pharmaceutical Federation UNESCO-UNITWIN Global Pharmacy Education Development Network is another example of a twinning project. It supports the development of professional competence in the exchange of experience and knowledge between universities and other learning institutions. Additionally, it supports collaborative practice between university teachers, researchers and administrators and other healthcare workers. The Network created its first global centre of excellence: the African Centre of Excellence in Pharmacy Education, with Ghana, Namibia, Nigeria, Uganda and Zambia as founding partners. One of the Network’s projects to improve educational outcomes related to research was to provide “lab boxes” to the schools lacking equipment. Another project is “PharmAcademy” which is a community site designed to connect and share knowledge and resources for pharmacy educators globally.

The Training for Health Equity Network, a north-south and south-south collaboration of schools striving to increase their social accountability, is focusing on joint research, sharing tools and resources, capacity development and advocacy. It has developed an evaluation framework [48] and is currently conducting cross-institutional, longitudinal studies on the location and career choices of the schools’ graduates, the health and social impact of its member schools in the Philippines as well as designing a return on investment study.

## Discussion

The evidence for return on investment in health is sound [49]. According to Jamison et al., 24 % of growth in full income in middle- and low-income countries from 2001 to 2011 grew out of improvements in health [50]. Investing in the workforce also provides returns. The health workforce remains a solid source of job creation and contributed up to 5 % of economic output during the recent economic crisis in Europe [51]. In Australia, a recent study shows that investing one Australian dollar (AUD) in primary care in remote indigenous communities could save between AUD 3.95 and 11.75 in public hospital expenses, in addition to the health and social benefits for patients [52].

However, despite some improvement, education of health workers remains chronically underfunded in many countries [4]. While more research and evaluations are required to strengthen the evidence base for what works and how, sufficient investment in health workforce education that is linked to multi-sectoral interventions and partnerships with stakeholders including local communities is needed [53]. The development of a fit for purpose workforce, advancement of primary care approaches, emphasis on the social determinants of health and a strong community focus all contribute to a world where transformational education can progress community health towards achieving health equity [54].

## Conclusions

Health workforce education that is socially accountable, aligned to meet the needs of the societies served and uses a collaborative approach to define and meet those needs, promises to be a key mechanism to maximize the impact of educational investments. The illustrative examples provided in this review from around the globe demonstrate numerous positive impacts including:

- Distributed community-engaged learning: Education occurs in or near underserved communities, resulting in economic and social benefits for those communities.
- Curriculum aligned with health needs: The health and social needs of the targeted; thus, communities guide education, research and service programmes, and graduates are thus better prepared to address them.
- Fit for purpose workers: Community needs are met through the embedding of education into health systems and increased revenue streams.
- Gender and social empowerment: Diversity and social empowerment in health professional education helps not only empower those professionals but can empower the communities these professionals serve.
- Interprofessional training: Reduction in costs and waste and improved patient-centred care.
- Collaborative partnerships: Sharing of research, tools and ideas to align competencies and maximize resources.

While more research is needed to understand these complex dynamic interventions and broader impact of specific and combinations of strategies to explore causality pathways, there is clearly a need to build a workforce that is responsive and accountable for meeting evolving needs of the communities they serve [4, 14].

## Abbreviations

AMTC, accelerated medically trained clinicians; ADZU, Ateneo de Zamboanga School of Medicine; AHSA, accredited social health activist; CHW, community health workers; IPE, interprofessional education; MEPI, Medical Education Partnership Initiative; NEPI, Nursing Education Partnership Initiative; NOSM, Northern Ontario School of Medicine; SA, socially accountable; UNM, University of New Mexico Health Sciences Center
